# Fatal hemoptysis despite complete remission of squamous cell carcinoma after chemoradiotherapy followed by combined ipilimumab and nivolumab: An autopsy case report

**DOI:** 10.1097/MD.0000000000046756

**Published:** 2025-12-26

**Authors:** Kazuhito Horie, Sohei Nakayama, Akiyoshi Hoshino, Masahiko Okada, Hideki Terai, Yusuke Suzuki, Takanori Asakura

**Affiliations:** a Division of Pulmonary Medicine, Department of Medicine, Keio University School of Medicine, Tokyo, Japan; b Department of Respiratory Medicine, Kitasato University Kitasato Institute Hospital, Tokyo, Japan; c Department of Pathology, Kitasato University Kitasato Institute Hospital, Tokyo, Japan; d Keio Cancer Center, Keio University School of Medicine Tokyo, Tokyo, Japan; e Department of Clinical Medicine (Laboratory of Bioregulatory Medicine), Kitasato University School of Pharmacy, Tokyo, Japan.

**Keywords:** cavitary lesion, chemoradiotherapy, hemoptysis, immune checkpoint inhibitor, lung cancer

## Abstract

**Rationale:**

Hemoptysis is a known complication of lung cancer that may occur after antiplatelet or anticoagulant therapy, chemotherapy, or antiangiogenic agents. Although immune checkpoint inhibitors have improved outcomes in lung cancer, hemoptysis remains a rare but potentially fatal adverse event. We describe an autopsy-confirmed case of complete tumor remission with cavitation leading to fatal hemoptysis after chemoradiotherapy (CRT) followed by combined ipilimumab and nivolumab therapy for squamous cell carcinoma.

**Patient concerns:**

A 68-year-old man with a history of heavy smoking was found to have a 70-mm right hilar lung lesion and was diagnosed with stage IIIC squamous cell carcinoma with 50% programmed death ligand-1 expression. After CRT and subsequent combination chemotherapy plus immune checkpoint inhibitors, he developed pneumonitis requiring prednisolone (PSL). During PSL tapering, he experienced fever and a new right upper lobe cavitary lesion, which enlarged despite empiric antibiotics. After discharge under apparently stable conditions on a reduced PSL dose, he suddenly developed massive hemoptysis.

**Diagnoses:**

The primary diagnosis was stage IIIC squamous cell carcinoma of the lung. During the treatment course, he developed immune checkpoint inhibitor–related pneumonitis and a new cavitary lesion in the right upper lobe. Autopsy revealed a necrotic cavitary lesion with vascular destruction in the right upper lobe, dense adhesions between the lung and chest wall, and no residual carcinoma, infection, or distant metastases, confirming complete tumor remission and fatal hemoptysis due to treatment-related vascular injury.

**Interventions:**

The patient received definitive CRT with carboplatin and paclitaxel plus 60 Gy thoracic radiotherapy, followed by carboplatin and paclitaxel combined with ipilimumab and nivolumab, and subsequently maintenance ipilimumab and nivolumab. Immune-related pneumonitis was treated with systemic PSL, which was tapered and subsequently re-escalated when fever and a new cavitary lesion appeared. Empiric broad-spectrum antibiotics were also administered.

**Outcomes:**

CRT and combined immune checkpoint inhibitor therapy achieved complete tumor remission pathologically. However, the progressive cavitary change in the irradiated lung ultimately resulted in massive hemoptysis, cardiac arrest, and death. Autopsy confirmed a necrotic cavity with vascular destruction without residual malignancy or infection.

**Lessons:**

In patients receiving ipilimumab and nivolumab after thoracic CRT for centrally located squamous cell carcinoma, rapid tumor necrosis and cavitation within irradiated lung with fragile vasculature may markedly increase the risk of severe and fatal hemoptysis. When initiating or resuming combined immune checkpoint inhibitor therapy in this setting, clinicians should carefully consider CRT-associated vascular fragility and potential immune-mediated vascular injury, and ensure close imaging follow-up and airway surveillance.

## 1. Introduction

Hemoptysis can occur in infectious and neoplastic conditions such as tuberculosis, bronchiectasis, and lung cancer. Although it often resolves spontaneously, approximately five percent of cases progress to an active, life-threatening state, with the potential for fatal massive hemoptysis.^[1,2]^ In patients with lung cancer, antiplatelet and anticoagulant therapies, cytotoxic chemotherapy, histological subtypes, and tumor location can contribute to hemoptysis.^[2]^ Antiangiogenic agents such as bevacizumab and ramucirumab may cause hemoptysis as an adverse event, and their use is contraindicated in patients with a history of hemoptysis. Moreover, previous reports have suggested a higher risk of massive hemoptysis when squamous cell carcinoma (SCC) lesions are located adjacent to major vessels.^[3]^ Therefore, appropriate risk assessment and treatment selection are crucial for overall decision-making in lung cancer care.

Immune checkpoint inhibitors (ICIs) targeting cytotoxic T-lymphocyte-associated antigen-4 and programmed death-1/programmed death-ligand 1 are increasingly used to treat lung cancer. Neoadjuvant chemotherapy, followed by surgical resection, is performed in patients with clinical stage III lung cancer when the tumor is considered resectable.^[4]^ In contrast, chemoradiotherapy (CRT) is the standard initial treatment for unresectable tumors, and durvalumab consolidation after CRT is an established standard of care with a durable 5-year survival benefit.^[5]^ However, if recurrence is diagnosed after CRT, systemic chemotherapy following the treatment principles of stage IV disease may be administered, with ICIs being a viable therapeutic option.

Interstitial pneumonia is a relatively common adverse event associated with ICIs and is categorized as an immune-related adverse event (irAE) that requires careful attention. Several reports have described fatal hemoptysis in patients with lung cancer after ICIs therapy.^[6,7]^ The potential causes of ICI-related hemoptysis include rapid tumor shrinkage with exposure of the central vessels and immune-mediated endothelial injury/vasculitis, which predispose patients to bleeding.^[6,8]^ Hemoptysis is a rarely reported adverse effect of ICIs in lung cancer populations.^[9]^ In fact, there was no clear signal of increased hemoptysis with ipilimumab and nivolumab compared to platinum-doublet chemotherapy.^[10]^ Moreover, in clinical trials of ipilimumab and nivolumab administered with stereotactic body radiotherapy, only a single case of fatal hemoptysis has been described, and details, including pathology, have not been reported.^[11]^ In contrast, multiple trials of ipilimumab and nivolumab after CRT have raised concerns regarding increased pneumonitis and treatment-related death despite promising efficacy signals.^[12,13]^

Despite case reports of post-ICI hemoptysis, autopsy-level evidence delineating the mechanisms after CRT remains limited, particularly regarding whether post-CRT vascular fragility and subsequent cavitation under ICIs contribute to fatal bleeding in the absence of a residual tumor. Herein, we report an autopsy-confirmed case of SCC treated with CRT followed by ipilimumab and nivolumab. The patient achieved complete pathological remission yet developed cavitation and fatal hemoptysis, which may inform future mechanistic and safety studies.

## 2. Case presentation

**Figure 1.**
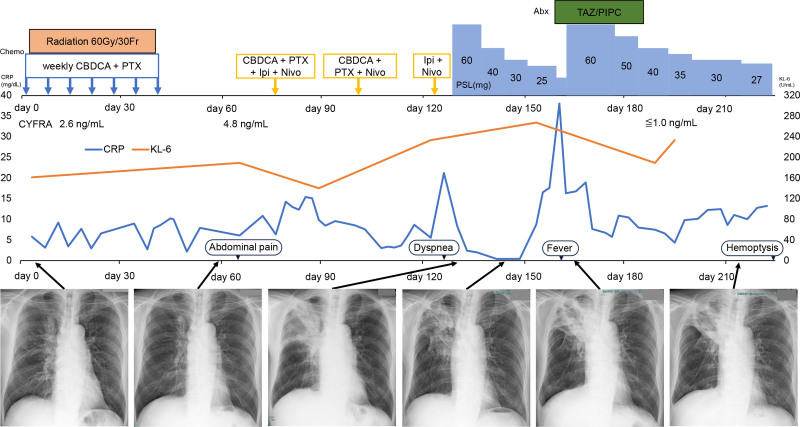
Clinical course of the patient. Abx = antibiotic, CBDCA = carboplatin, Chemo = chemotherapy, CRP = C-reactive protein, CYFRA = cytokeratin 19 fragment, Ipi = ipilimumab, KL-6 = krebs von den Lungen-6, Nivo = nivolumab, PSL = prednisolone, PTX = paclitaxel, TAZ/PIPC = tazobactam/piperacillin.

**Figure 2.**
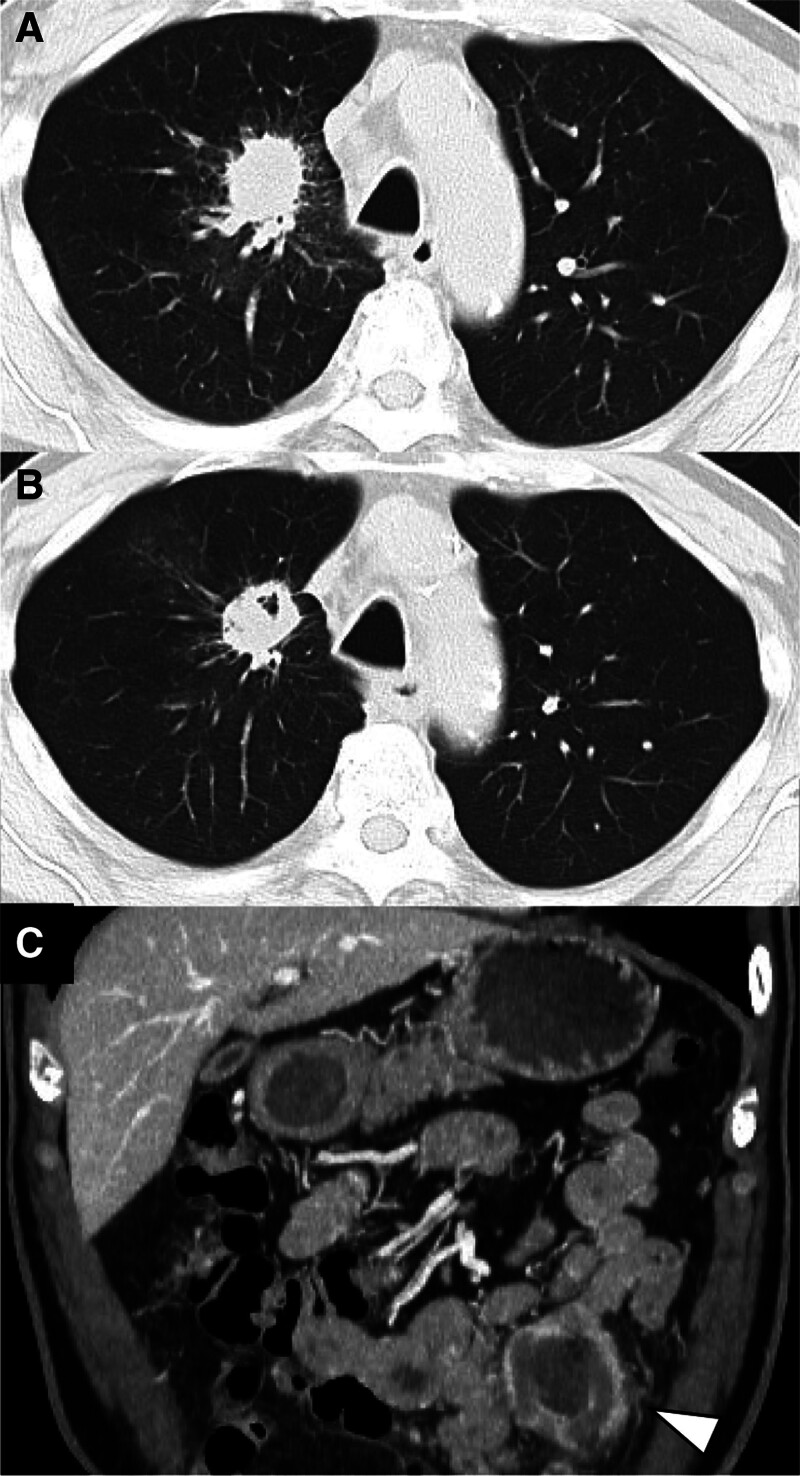
(A) Initial chest computed tomography (CT) image showing a 70 mm lesion in the right pulmonary hilum. (B) Chest CT image after completion of chemoradiotherapy (CRT) showing tumor reduction. (C) Abdominal CT image obtained two weeks after completion of CRT showing thickened small-bowel walls (arrowhead), considered suspicious for metastasis.

Figure 1 summarizes the patient’s clinical course. A 68-year-old man was referred to our hospital with an abnormal chest shadow. He had a history of hypertension, diabetes, and a 60 pack-year smoking history. Upon examination, a 70 mm lesion was identified in the right hilum of the lungs (Fig. 2A). Pathological examination of the transbronchial biopsy specimen revealed SCC. A 18F-fluoro-deoxyglucose positron emission tomography/computed tomography (CT) showed no distant metastases but showed accumulation in the right supraclavicular lymph node, indicating clinical stage IIIC (cT4N3M0) SCC. No actionable driver mutations were identified, and PD-L1 expression in the tumor was 50%.

The patient underwent CRT (weekly carboplatin 200 mg and paclitaxel 70 mg with radiation therapy of 60 Gy/30 Fr) (day 5). The lesion in the right hilum had decreased in size after CRT (Fig. 2B). Two weeks after completion of CRT, the patient developed abdominal pain and distension. Contrast-enhanced CT revealed thickening of the small bowel wall, with additional findings suggestive of peritoneal dissemination (Fig. 2C). Although histological confirmation was not obtained, there was no improvement with nasogastric decompression and bowel rest with intravenous fluids, and the imaging findings favored metastatic disease. Therefore, secondary therapy with carboplatin 560 mg, PTX 330 mg, ipilimumab 60 mg, and nivolumab 360 mg was administered (day 75), which improved the abdominal symptoms.

**Figure 3.**
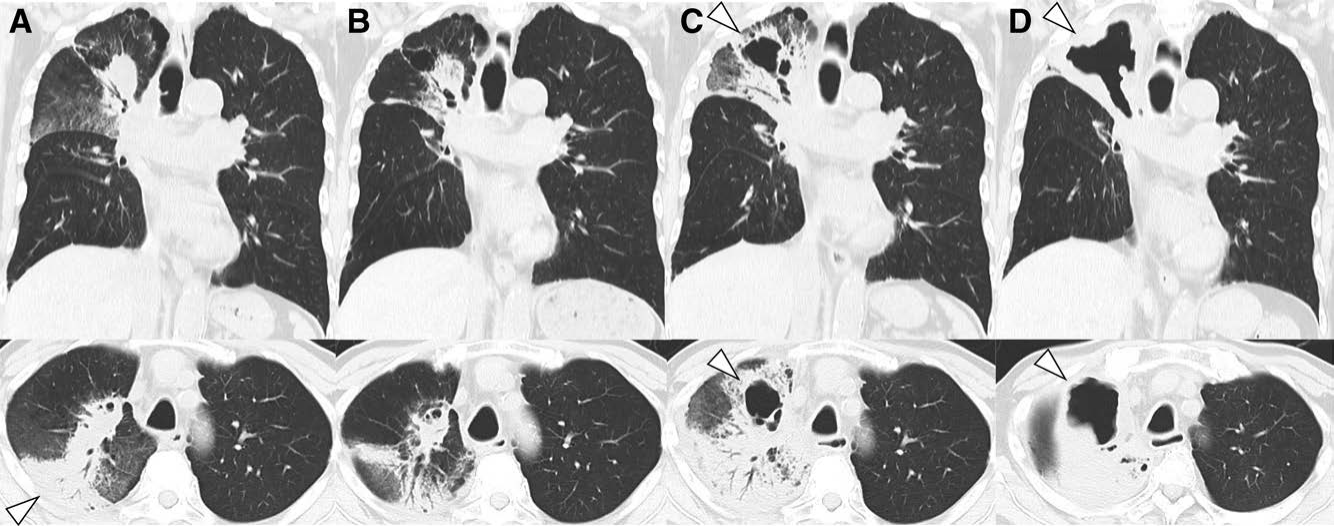
(A) Chest CT image after maintenance therapy with ipilimumab showing consolidation in the right upper lobe (arrowhead), raising suspicion for radiation/immune-related pneumonitis. (B) Improvement of consolidation after administering prednisolone (PSL) at 60 mg/d. (C) CT findings upon PSL reduction to 25 mg/d showing worsening infiltration with a cavitary lesion in the right upper lobe (arrowhead). These findings suggested relapse of pneumonia, either infectious or radiation/immune-related pneumonitis, prompting initiation of TAZ/PIPC and re-escalation of PSL. (D) Increased PSL to 60 mg/d and using antibiotic resulted in consolidation improvement; however, the cavitary lesion in the right upper lobe continued to enlarge (arrowhead).

After 2 cycles of second-line therapy, maintenance ipilimumab (60 mg) every 6 weeks and nivolumab (360 mg) every 3 weeks were initiated on day 123. On day 129, cough and dyspnea appeared, along with deterioration of the shadow in the right upper lobe (Fig. 3A), suggesting radiation/immune-related pneumonitis. Treatment with prednisolone (PSL, 60 mg/d) improved the symptoms and shadows (Fig. 3B). PSL dose (60 mg/d) was tapered weekly to 40 mg (day 137) and 30 mg (day 145). Upon tapering PSL to 25 mg/d, the patient developed fever with markedly elevated C-reactive protein (37.99 mg/dL; reference < 0.14 mg/dL). Chest CT performed on day 164 revealed worsening pulmonary infiltrates and a cavitary lesion in the right upper lobe (Fig. 3C). At that time, abdominal CT demonstrated resolution of the previously noted small bowel wall thickening and disappearance of the findings suspicious for peritoneal dissemination.

The PSL dose was increased to 60 mg/d, and tazobactam/piperacillin was administered. As the C-reactive protein level decreased, the size of the cavitary lesion in the right upper lobe increased (Fig. 3D). The sputum culture was negative and serum β-D-glucan was normal (8.7 pg/mL, range < 20 pg/mL), *Aspergillus* antigen was negative. The patient was discharged when the PSL dose was reduced to 27 mg/d and in a stable condition. However, 25 days after discharge, the patient was transported due to cardiac arrest and massive hemoptysis. Despite temporary cardiac resuscitation, the patient died the same day (day 230).

**Figure 4.**
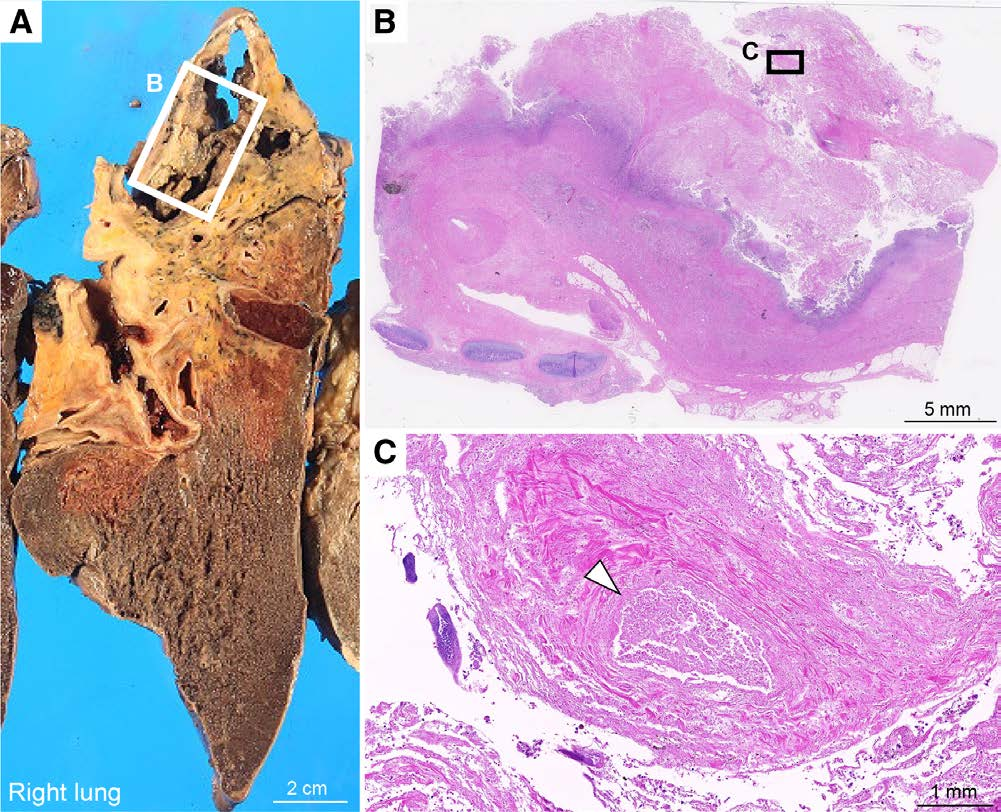
(A) Gross pathology showing a macroscopic view of the right lung at autopsy without evidence of carcinoma, consistent with complete remission. (B) Hematoxylin and eosin (H&E) staining showing only organized necrotic tissue within the lung. (C) Scattered blood vessels in the necrotic tissue (arrowhead).

An autopsy was performed and macroscopic examination revealed severe adhesions of the right upper lobe of the lung to the chest wall with necrotic cystic lesions and blood vessel structures (Fig. 4A). Microscopic examination revealed scattered blood vessels within organized necrotic tissue in the lungs (Fig. 4B and C), which may be associated with massive hemoptysis. There was no evidence of SCC. Cultures obtained from the cavity wall and blood clots in the right upper lobe yielded *Pseudomonas aeruginosa*, whereas sputum cultures obtained at the time of temporary cardiac resuscitation did not. There was no evidence of fungal infection. None of the organs, including the small intestine, showed evidence of carcinoma, indicating complete response.

## 3. Discussion

This case report describes a patient with stage IIIC SCC who developed a lesion suspicious of small bowel metastasis after CRT and subsequently received ipilimumab and nivolumab in combination with platinum-based chemotherapy. The patient developed radiation pneumonitis and immune-related pneumonitis, which progressed to cavitary changes, and ultimately experienced fatal massive hemoptysis. The autopsy revealed no evidence of malignancy in the lungs or other regions, including the small intestine, indicating complete remission. This clinical course suggests that ipilimumab and nivolumab exert potent antitumor effects yet may precipitate potentially fatal complications. Particularly, the interaction between CRT-induced vascular fragility and ICI-driven immune activation may contribute to the risk of cavitation and hemoptysis.

IrAEs can affect multiple organs, including the lungs, endocrine system, and skin. Although the precise molecular mechanisms remain incompletely defined, several hypotheses have been proposed^[14,15]^: ICIs inhibit molecules that regulate costimulatory signals, expanding and activating the T-cell repertoire. While this augments antitumor immunity, it may also activate T cells against antigens shared by cancer and normal tissues. ICIs may affect B cells and induce autoantibody production, potentially unmasking or exacerbating latent autoimmunity.

In the treatment of lung cancer, the occurrence of irAEs with ICIs has been associated with improved survival outcomes, with patients who develop irAEs showing significantly longer progression-free survival (PFS) and overall survival (OS) than those without irAE.^[16]^ It has also been reported that pulmonary toxicity occurs more frequently with ICIs therapy in lung cancer than in other tumor types, with approximately 10% of lung cancer patients experiencing pulmonary toxicity, particularly among smokers and those with preexisting interstitial lung disease.^[17]^ In contrast, endocrine and dermatological irAEs have been associated with prolonged PFS/OS, whereas such prolongation has not been consistently observed for pulmonary irAE.^[18]^

In stage IIIC disease, durvalumab maintenance after CRT is the current standard of care; however, while the risk of low-grade pneumonitis is recognized with post-CRT durvalumab, an excess risk of severe pneumonitis has not been consistently observed.^[19]^ Several strategies have been explored for consolidation after CRT in patients with unresectable stage III non-small cell lung cancer (NSCLC). In one trial, patients received concurrent CRT with nivolumab, followed by maintenance therapy with either ipilimumab plus nivolumab or nivolumab alone, and the outcomes were compared with durvalumab maintenance.^[12]^ No improvement in PFS and OS were observed compared with durvalumab, whereas the incidence of grade ≥ 3 adverse events (including pneumonitis) and treatment-related deaths was higher with ipilimumab plus nivolumab than with durvalumab monotherapy. Another study comparing post-CRT ipilimumab and nivolumab with post-CRT durvalumab reported favorable PFS and OS signals for the combination, but at the cost of higher irAE rates.^[13]^ Although hemoptysis was not specifically reported in these trials, the data suggest increased treatment-related toxicity of ipilimumab and nivolumab after CRT. As potential mechanisms for higher adverse event rates after CRT to ICIs, we considered the following hypotheses: CRT remodels the tumor microenvironment with increased CD8^+^ T-cell infiltration^[20]^; tumor immune escape may be accompanied by an expanded exhausted T-cell compartment^[21]^; and ICIs administration may reinvigorate exhausted T cells, enhancing antitumor activity, but also potentially increasing immune-mediated toxicities.

Massive hemoptysis in this case most likely arose from bleeding within the cavitary lesion. Rupture of the exposed intralesional vessels is plausible, with CRT and ICIs potentially acting as cofactors. Cavitary lesions are associated with an increased risk of hemoptysis, and patients with NSCLC and cavitation have been reported to bleed more frequently.^[2,22]^ In stage III NSCLC, tumor cavitation after CRT has been described; 18% of patients in a single-institution cohort developed cavitation, and these patients showed relatively frequent severe pulmonary complications, including fatal hemorrhage, highlighting the need for careful management.^[23]^ Moreover, thoracic irradiation can induce endothelial injury, inflammation, and fibrosis, leading to vascular remodeling and fragility, which may contribute to the risk of bleeding in this setting.^[24,25]^ Although hemoptysis is considered a rare adverse event of ICIs, there have been case reports of ICIs-associated hemoptysis.^[6,7,26]^ Furthermore, in bevacizumab-containing chemotherapy, the addition of ICIs has been associated with a higher incidence of hemoptysis in large trials on non-squamous NSCLC.^[27]^ The bleeding risk was particularly notable in tumors with preexisting cavitation or proximity to major vessels, clinical contexts already recognized as high risk for pulmonary hemorrhage.^[2]^

Several studies have also documented marked tumor shrinkage followed by cavitation after ICIs treatment, sometimes in the setting of high PD-L1 expression; in rare cases, this sequence has culminated in fatal hemoptysis. One possible mechanism is that the interaction between PD-L2 and the repulsive guidance molecule, BMP co-receptor B, in the alveolar epithelium triggers excessive tissue damage responses mediated by activated T cells during ICIs therapy.^[28,29]^ In addition, although vasculitis is an uncommon irAE, ICI-associated small- and large-vessel vasculitides have been reported and can manifest as diffuse alveolar hemorrhage; thus, immune-mediated vascular fragility represents another plausible contributor in selected patients.^[30]^ Taken together, these observations suggest multiple non-mutually exclusive pathways – therapy-induced necrosis with cavitation, vascular involvement, and local immune dysregulation – through which ICIs may, in rare instances, be linked to hemoptysis.

Infections are a common cause of hemoptysis. In the literature, a 68-year-old man with SCC developed infiltrative and cavitary lesions and was diagnosed with invasive aspergillosis during durvalumab maintenance after CRT; due to uncontrolled infection, he underwent lung resection, and pathology revealed no residual tumor, but Aspergillus colonies.^[26]^ In contrast, our patient showed no evidence of an Aspergillus infection. Although *P. aeruginosa* was isolated from the cavity wall and intralesional clots, suggesting a possible superimposed secondary infection, negative sputum cultures, and progressive cavity enlargement despite antibiotic therapy leave the degree of infectious contribution uncertain. Furthermore, infection (including fungal etiologies) is a well-recognized driver of hemoptysis in lung cancer and should remain in the differential diagnosis when new cavities appear under post-CRT ICIs therapy.^[1]^

As a limitation of the present case discussion, the possibility of small bowel metastasis was determined solely on clinical grounds, without histopathological confirmation. At autopsy, immunohistochemical evaluation of the endothelium/vascular wall, elastic fiber staining, and vascular reconstruction were not performed; therefore, the quantitative assessment of vascular injury was limited. Furthermore, fluctuations in the corticosteroid dose and antibiotic use may have influenced the disease course, making a clear causal attribution difficult.

## 4. Conclusion

In conclusion, although amplification of tissue destruction by secondary infection cannot be excluded, the fatal hemoptysis in this case was most likely the result of a composite mechanism: therapy-induced necrosis with cavitation, leading to exposure and rupture of vessels within or adjacent to the cavity, and CRT-associated endothelial injury and vascular remodeling, which increased the propensity for bleeding.

Clinicians should consider radiotherapy-induced changes in the tumor microenvironment, weigh potential adverse events related to excessive immune activation, and carefully consider PD-L1 expression and overall toxicity risk before initiating ICIs after CRT.

## 5. Patient perspective

We initially believed that an effective treatment would allow us to return to everyday life. After CRT, the care team recommended immune checkpoint therapy, and consent was obtained after discussing the potential benefits and risks. During treatment, we managed medications and frequent visits; when his breathing eased, we felt hopeful, yet new imaging findings and fever caused anxiety.

After discharge, we tried to structure daily life around fluctuating energy levels and contacted the team whenever concerns arose. The sudden massive hemoptysis was devastating. Although we wondered if we could have noticed the warning signs earlier, we are grateful for the clear explanations and the team’s efforts. For families in similar situations, we should emphasize understanding the treatment plan together, knowing what changes at home should prompt urgent contact, and not hesitating to seek help early. (This account was reviewed and approved by the family for publication).

## Acknowledgments

Portions of this manuscript were assisted by OpenAI’s ChatGPT, which was used to help with language editing and phrasing. The authors take full responsibility for the content of the manuscript.

## Author contributions

**Conceptualization:** Kazuhito Horie, Takanori Asakura.

**Investigation:** Kazuhito Horie, Akiyoshi Hoshino, Takanori Asakura.

**Supervision:** Sohei Nakayama, Akiyoshi Hoshino, Masahiko Okada, HIdeki Terai, Yusuke Suzuki, Takanori Asakura.

**Writing – original draft:** Kazuhito Horie.

**Writing – review & editing:** Sohei Nakayama, Masahiko Okada, Hideki Terai, Yusuke Suzuki, Takanori Asakura.
